# Supplementary material for: Additive Effects of Solid Paraffins on Mechanical Properties of High-Density Polyethylene
Source: Polymers (Basel). 2023 Mar 6;15(5):1320. doi: 10.3390/polym15051320 (PMC10007512; doi:10.3390/polym15051320)
Supplement: Supplementary file 1 [file polymers-15-01320-s001.zip › polymers-2207953-supplementary.pdf]

Figure S1 shows the temperature dependences of the storage modulus *E'*, loss modulus *E"* and loss tangent tan δ measured at 10Hz and a heating rate of 2 °C/min for HDPE/nPW and HDPE/iPW with PW contents of 10w% and 5w%.

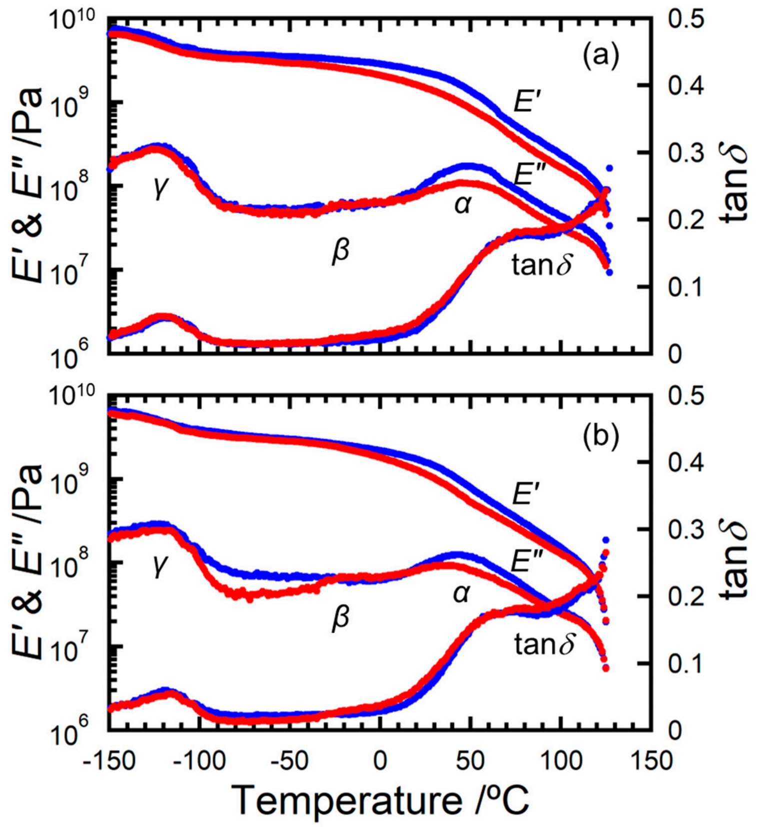

Figure S1. Dynamic viscoelastic spectra of (a) HDPE/nPW (95w/5w) (blue) and HDPE/iPW (95w/5w) (red) and (b) HDPE/nPW (90w/10w) (blue) and HDPE/iPW (90w/10w) (red).

Figure S2 shows the stress-strain curves measured at an elongation speed of 10 mm/min for HDPE/iPW blends. The stress values were normalized by the content of HDPE.

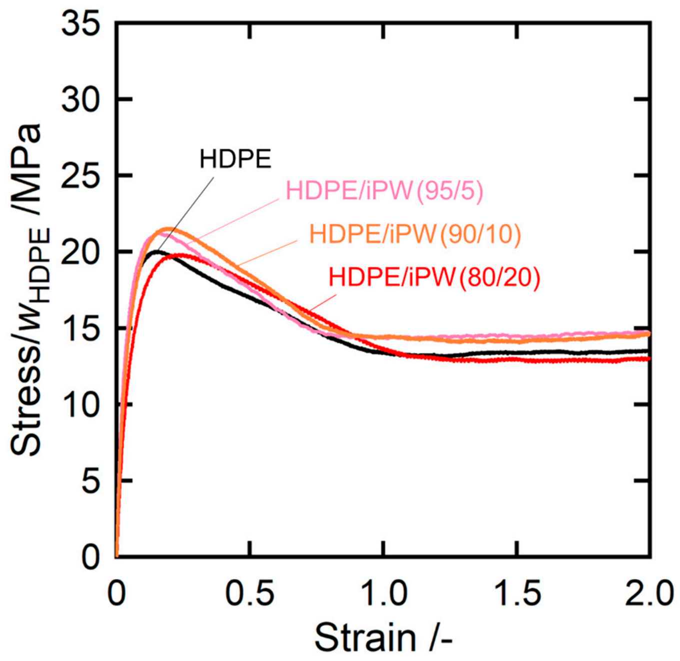

Figure S2. Stress–strain curves of initial HDPE and HDPE/iPW. The stress values are divided by the weight fraction of HDPE.
